# Unpredictable Outcomes of a Regenerative Endodontic Treatment

**DOI:** 10.1155/2021/2478310

**Published:** 2021-10-06

**Authors:** Zahra Mohammadi, Hadi Assadian, Behnam Bolhari, Mohammadreza Sharifian, Mehrfam Khoshkhounejad, Nazanin Chitsaz

**Affiliations:** Department of Endodontics, School of Dentistry, Tehran University of Medical Sciences (TUMS), Iran

## Abstract

Regenerative endodontic treatment (RET) is a valuable treatment for necrotic immature teeth with many advantages such as increasing root length and thickness of root wall. The success of RETs is based on healthy stem cells, suitable scaffolds, and growth factors and takes place when bacterial contamination is well controlled. The aim of this article is to address controversy in a case with multiple success criteria. This paper reports a 9-year-old boy with a complicated crown fracture of the maxillary left central incisor about three years prior to referral with a diagnosis of intrusive luxation with spontaneous reeruption. The tooth had an underdeveloped root and a well-defined periapical radiolucent lesion around the root apex. RET was considered according to the stage of root development. Upon the three-week recall session, the clinical examination indicated that the patient was asymptomatic in the affected site. However, the patient returned two weeks later with a sinus tract pertaining to the apex of tooth #9. Therefore, debridement of the root canal space was repeated and the RET redone. On the second trial, the patient was symptom-free, but no more evidence of root maturation was observed on 18-month follow-up. The tooth was asymptomatic (without swelling, drainage, and pain) during this time, and esthetics was provided for the patient.

## 1. Introduction

All endodontic treatments are aimed at prevention or treatment of apical periodontitis (AP). The goal for treatment of immature permanent teeth is to restore the physiologic structures and functions of the pulp dentin complex [1]. As a substitute to conventional root canal treatment, regenerative endodontic treatment (RET), is a valuable treatment for necrotic immature teeth with many advantages such as increasing root length and thickness of root walls unlike other treatments such as apical plug or apexification with calcium hydroxide [2–4].

The success in RETs relies on the tripod of healthy stem cells, suitable scaffolds, and growth factors [5–7] and takes place when bacterial contamination is eliminated from the environment [8]. The successful regenerative endodontic procedure is measured via increased root length, thickened canal walls, and closure of the root apex and even in some cases with positive responses to pulpal sensibility tests [9]. Several published cases with immature roots indicate that the RET has the potential to encourage continued maturation of the root in terms of width and length. This procedure includes competent infection control, a suitable scaffold for fresh tissue ingrowth, and adequate coronal seal [10]. Achievement of success in RETs requires no signs and symptoms of disease such as pain, swelling, or sinus tract, as well as radiographic evidence of periapical healing with increased root length and root canal wall thickness, indicating continued root development [11]. Unhealed periapical lesions, fractures, and failures to induce periapical bleeding are the main causes associated with RET failure [12].

There are several factors which affect the outcome of RET such as apex diameter, patient age, and degree of root canal infection [13].

Histological examinations of the nature of the tissue formed in humans and animals showed a tissue similar to cementum and osteodentin [14–16] and a soft tissue similar to fibrous connective tissue containing vessels [15, 17].

The success of RETs is dependent on the patient's potential to heal the dental pulp tissues. The provision of RETs should be restricted to healthy patients who can heal and benefit from the procedure [18]. The survival of cells and regeneration of tissues is sensitive to the conditions within the intracanal environment. The materials should be biocompatible, because they can cause cell death and allow bacterial leakage [19].

There are various guidelines and studies that have defined the success of RET more precisely.

Diogenes et al. [20] describe the RET outcome in three levels: patient-based outcome (absence of swelling, drainage, pain, tooth survival and function, and tooth esthetics); clinician-based outcome (radiographic healing, radiographic root development, and positive vitality responses); and scientist-based outcome (histologic evidence of complete regeneration) are taken into account.

Staffoli et al. [18] suggested treatment success as healing of apical radiolucency within an average of 8 months and root development within 18 months after treatment and stated that absence of these radiographic features within 2 years or the presence of signs and symptoms equals treatment failure. American Association of Endodontics (AAE) [21] categorized success rates of RETs as primary, secondary, and tertiary goals, with elimination of symptoms and bone healing as primary, root maturation as secondary, and positive response to vitality testing as the tertiary goals. European Society of Endodontology (ESE) [22] describes success of regenerative endodontic procedure as lack of signs and symptoms, discoloration, and patient's acceptance and radiographic detection of new PDL along the inner wall of root canal.

Even though the primary goal of healing AP and continued root development is mostly reached in REPs, the unpredictability of the regeneration evidence from histological point of view is a shortcoming. It is completely comprehensible that achieving this goal is very demanding due to its multifactorial nature [23].

Most studies in regenerative endodontics are limited to successful cases with an increase in root length or closure of the apex [24–26], while cases that are limited to relieving the patient's symptoms have been neglected, although these treatments can be categorized as successful in the patients' view [20].

The aim of the current case report was to describe clinical RET using calcium hydroxide mixed with chlorhexidine as intracanal medicament for a patient's postoperative functional teeth without any signs of root development on 18-month follow-up.

## 2. Case Report

**Figure 1:**
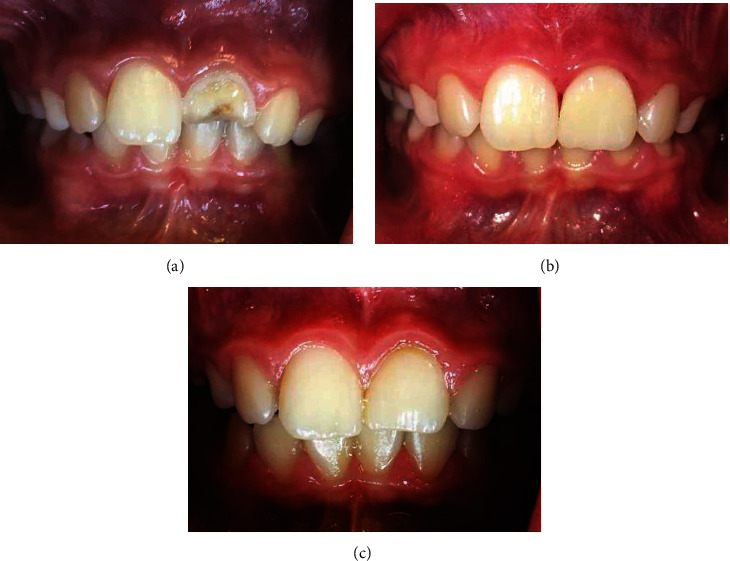
Intra-oral photograph of the fractured maxillary left central incisor in a 9-year-old boy (a) preoperative image, (b) 3-month recall, and (c) 18-month recall.

The patient was a 9-year-old boy referred by a general dentist to the School of Dentistry, Tehran University of Medical Sciences (TUMS), Tehran, Iran, in 2018. His medical history revealed a systemically healthy patient categorized as class I according to ASA health status. His dental history indicated a complicated crown fracture of the maxillary left central incisor about three years ago due to a fall. The patient was admitted to the Department of Endodontics with a prereferral diagnosis of intrusive luxation with spontaneous reeruption and complicated crown fracture by a general dentist. Extraoral examination showed no scar in the pertinent soft tissues. Upon intraoral examination, complicated crown fracture of tooth #9 was noted (Figure 1(a)). The patient's oral hygiene was good.

**Figure 2:**
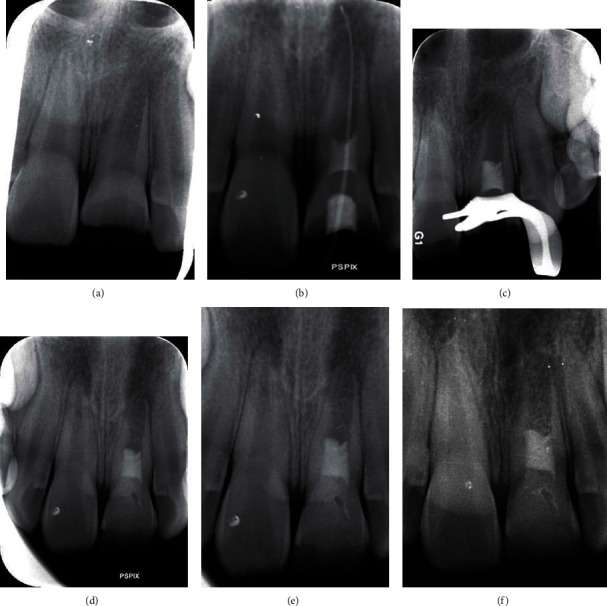
The radiographic images showing (a) the initial preoperative condition, (b) tracing the mucosal sinus tract with a gutta percha point, (c) second try, (d) 3-month recall, (e) 9-month recall, and (f) 18-month recall.

A pulp sensitivity test to cold stimulus using CO_2_ snow spray (Luber cool, Luber, Germany) showed no response in the involved teeth. The periodontal examinations revealed normal conditions (i.e., probing < 3 mm, mobility < 1 mm, and insensitive to percussion/palpation). Periapical radiographic examination showed that tooth #9 had an open apex shorter root and thinner radicular walls in comparison with the adjacent teeth. A well-defined periapical radiolucent lesion was evident around the root apex (see Figure 2(a)). The maxillary left central incisor was diagnosed as having a necrotic pulp with asymptomatic apical periodontitis. RET was considered according to the stage of root development. The patient's parents were informed about the treatment process, and informed consent was provided. In the first session, local anesthesia was administered using 2% lidocaine with 1 : 80,000 epinephrine (Daru Pakhsh, Tehran, Iran), and the access cavity was prepared under rubber dam isolation. The working length was determined using an electronic apex locator (Root ZX, J. Morita Corp., Tokyo, Japan) and confirmed radiographically. Then, chemical disinfection was performed using 20 mL of 1.5% sodium hypochlorite (Hypo-EndoX, Morvabon, Iran) with passive ultrasonic activation using a piezoelectric ultrasonic unit (Varios 970, NSK, Japan), with the power setting at 10, for 5 min. Then, calcium hydroxide (Golchadent, Tehran, Iran) was applied into the canal in a creamy consistency, using a #15 K-file (MicroMega, Besangon, France) placed 2 mm short of the working length. Then, the access cavity was sealed with reinforced zinc-oxide eugenol cement (Zoliran, Golchadent, Iran). Upon the three-week recall session, the clinical examination indicated that the patient was asymptomatic in the affected site. Local anesthesia was administered using infiltration of 3% mepivacaine plain (Daru Pakhsh, Tehran, Iran), and the tooth was isolated with rubber dam. The remnants of the intracanal medicament were removed using passive ultrasonically activated irrigation (Varios 970, NSK, Japan) with 20 mL normal saline solution. The canal was irrigated with 20 mL of 17% EDTA solution for 5 min and then dried with sterile paper points. Blood was harvested from patient's brachial artery, and PRF was provided using a centrifuge (DUO Quattro, Process for PRF, Nice, France) at 208 g for 8 min. Bleeding was induced by a sterile manual #60 K-file (MicroMega, Besangon, France), within the canal. The intracanal PRF was placed 3 mm apical to the CEJ level. Dentin-bonding agent (Single Bond, 3M, ESPE, USA) was applied to the internal access cavity walls, and then, MTA Angelus (Angelus, Londrina, PR, Brazil) as a coronal barrier was applied using an MTA carrier. A moist cotton pellet was placed on top of the bulk of MTA, and the access cavity was sealed with reinforced zinc-oxide eugenol cement (Zoliran, Golchadent, Iran). It was scheduled to check MTA setting 1 day later, but the patient missed the appointment and returned two weeks later with a sinus tract. Upon radiographic evaluation, the sinus tract was traced with a #30/0.02 gutta percha point and its extension to the apex of tooth #9 was confirmed (see Figure 2(b)). Due to the presence of the draining sinus tract, debridement of the root canal space was repeated after injection of 2% lidocaine anesthesia and 1 : 80,000 epinephrine (Daru Pakhsh, Tehran, Iran), using 20 mL of ultrasonically (Varios 970, NSK, Japan) activating 1.5% sodium hypochlorite solution (Hypo-EndoX, Morvabon, Iran) under rubber dam isolation. Mechanical instrumentation was carried out using light circumferential filing with a #25 Hedstrom file (Dentsply Sirona, Ballaigues, Switzerland). Then, calcium hydroxide (Golchadent, Tehran, Iran) mixed with 2% chlorhexidine solution (Morvabon, Tehran, Iran) dressing was inserted into the root canal in a creamy consistency, and the tooth was temporarily filled with reinforced zinc oxide eugenol (Zoliran, Golchadent, Iran). The draining mucosal sinus tract healed after 10 days.

Therefore, after infiltration of 3% mepivacaine without a vasoconstrictor (Daru Pakhsh, Tehran, Iran), the dressing was removed similar to previous try under rubber dam isolation. The canal was rinsed with 20 mL of 17% EDTA solution (Sigma-Aldrich, St. Louis, MO, USA). PRF was prepared, and bleeding was induced by a sterile manual file as described above. The PRF, Dentin-bonding agent (Single Bond, 3M, ESPE, USA), and coronal barrier (Retro-MTA (bioMTA, Seoul, South Korea)) was placed as pervious try, access cavity sealed with reinforced zinc-oxide eugenol (Zoliran, Golchadent, Iran). (Figure 2(c)) After 1 week, the MTA was set and the crown was restored by composite resin (FGM, Joinville, Brazil). Follow-up sessions were planned. On recall visits, the tooth was asymptomatic and had a normal condition during periodontal tests. The follow-up radiographs and photographs are shown in Figures 1 and 2.

## 3. Discussion

In this case report, the patient had a history of dental trauma. After more than 18 months of recall, the teeth were in normal condition during periodontal tests and were still functional. In addition, the tooth functionality and esthetics were restored but no further root development was observed.

A recent study revealed that root development potential of immature necrotic teeth is related to the vitality of Hertwig's epithelial root sheath. Therefore, there might be a correlation between dental traumatic history and root development; the longer the duration of pulp necrosis, the lower the rate of root development after RET [27].

The patient represented a history of dental traumatic injury with possibly 3 years of pulpal necrosis. There is no evidence of root canal development in an 18-month recall.

In the classic RET protocol, passive decontamination is performed with sodium hypochlorite without conventional mechanical instrumentation for prevention of periapical cellular destruction. However, more recently mechanical instrumentation is used to wipe out necrotic pulp and remove the intracanal medicament [28].

A previous review claimed that immature teeth with an extended history of pulp necrosis had an unfavorable radiographic outcome after RET [29]. The contention of this argument is that even in the presence of some residual bacteria, traditional root canal treatment can result in the healing of periapical disease, but at the same way, it may not apply to RET [19]. Residual bacteria have a critical negative effect on the outcome of RETs [8]. Eradication of bacteria from the pulp canal has an important role in successful RET because revascularization halts in the presence of infection [17].

One approach for disrupting the biofilm is gentle brushing of the root canal wall with endodontic instruments (e.g., Hedstrom files) [30]. In case of persisting symptoms, repetition of the protocol using root canal disinfection by another regimen or the same drugs for a longer period of time is recommended [18].

In this case, on a second try, in addition to gentle brushing of the root canal walls, the intracanal medicament was used once more. Hence, 2% chlorhexidine was added to improve antimicrobial efficacy of calcium hydroxide. Correspondingly, de Jesus et al. reported successful treatment results with the use of calcium hydroxide mixed with chlorhexidine as an intracanal medicament in a patient with a past history of intrusive luxation and complicated crown fracture [31].

One of the limitations of regeneration in compromised teeth is the lack of space for intracanal posts. In the present case, due to the sufficient remaining enamel surface, direct composite build-up was performed. In immature permanent teeth with necrotic pulp when the pulp space is needed for a post/core in the final restoration, the modified apexification procedure has been suggested [32]; of course, it will be difficult to remove the MTA in case of treatment failure.

MTA prevents microleakage in addition to being biocompatible and promoting regeneration of the tissue when placed in contact with the dental pulp or periradicular, but one of the major problems with MTA, beside its setting time, is the very low value and unpredictable bonding to restorative materials. Therefore, delayed restoration improves bond strength [33]. Permanent restoration of the tooth in the present case was performed with composite resin after ensuring complete setting of MTA (1 week later).

Another shortcoming with using MTA as a coronal barrier is color change possibility in the medium/long term [34].

In order to prevent discoloration, dentin bonding was applied to seal the dentin tubules, in addition to limiting MTA placement under the CEJ level.

The results of the present study have shown that root development or increase in root canal wall thickness are not the only success criteria of treatment but also the tooth functionality and esthetic for young patients to give time for bone growth should be sufficient; so if the tooth becomes symptomatic in the future, the tooth site will be suitable for alternative treatments such as dental implants.

From this point of view, this case can belong to “primary success” according to AAE [21]. However, taking the ESE guidelines into consideration, the treatment was considered as “failed” due to the lack of root length increase [22]. Therefore, AAE criteria for success in RETs seem to be more comprehensive more specifically in border line cases.

There is also a strong need to report failed or ambiguous RET cases in endodontic literature besides successful ones to have a more accurate evaluation about RET rate of success.

## Data Availability

Almost all data are given and reviewed in the article, and if more data is needed, it will be sent to you from the corresponding author upon request.
